# New Insights into Adipose Tissue Metabolic Function and Dysfunction, 2nd Edition

**DOI:** 10.3390/ijms25179258

**Published:** 2024-08-27

**Authors:** Giovanni Pallio, Federica Mannino

**Affiliations:** 1 Department of Biomedical and Dental Sciences and Morphological and Functional Imaging, University of Messina, Via C. Valeria, 98125 Messina, Italy; 2 Department of Medicine and Surgery, University of Enna “Kore”, Contrada Santa Panasia, 94100 Enna, Italy; federica.mannino@unikore.it

The adipose organ is well recognized for its role in energy storage and mobilization, responding to nutrient availability, the body’s needs, and thermogenesis, thereby regulating the organism’s energy balance. Mammals have two main types of adipose tissue: white (WAT) and brown (BAT) adipose tissue. White adipocytes feature a single lipid droplet and a low number of mitochondria, while brown adipocytes, primarily found in the interscapular region, possess multilocular lipid droplets and significantly more mitochondria than white adipocytes. A third subtype, known as brite or beige adipocytes can be induced following the so-called browning process that transforms white-adipocyte-like to a brown adipocyte-like phenotype [1]. WAT, widely distributed throughout the body, plays a central role in modulating energy homeostasis in response to changes in systemic energy levels and is also involved in endocrine signaling, affecting various metabolic responses. Additionally, WAT secretes lipokines, adipokines, and exosome microRNAs, which impact a range of physiological processes [2,3]. Although BAT constitutes only a small portion of total adipose tissue, its capacity to induce adaptive thermogenesis has a significant metabolic impact. Emerging evidence also indicates that BAT plays a role in endocrine signaling by secreting various signaling molecules [4,5]. These metabolic and thermogenic responses are largely driven by the unique characteristics of the mitochondrial population. In fact, the mitochondria in white and brown adipocytes are functionally linked to the regulation of intracellular metabolism, with brown adipocyte mitochondria expressing high levels of uncoupling protein-1 (UCP1), which facilitates energy dissipation in thermogenic and metabolic adaptive responses. Like brown adipocytes, beige adipocytes express UCP1 and are highly active both metabolically and thermogenically, contributing to BAT’s role in regulating body temperature, energy homeostasis, and body weight control [6,7,8]. Additionally, all three adipocyte types have the potential to transdifferentiate into mammary luminal secretory epithelial cells, known as “pink adipocytes” (PAT), which are essential for milk secretion during pregnancy and lactation. Moreover, marrow adipose tissue (MAT) in bones is associated with yellow adipocytes. In healthy, lean individuals, MAT accounts for more than 10% of total fat mass. Yellow adipocytes, while similar in shape to white adipocytes, possess a distinct gene expression profile, lipid composition, and metabolism [9,10]. Importantly, all types of adipose tissue undergo finely tuned structural and metabolic remodeling in response to physiological stimuli. This plasticity ensures metabolic flexibility to meet the body’s needs, involving dynamic communication between adipocytes and various progenitor cells, immune cells, vascular cells, and sympathetic neurons, which, in turn, respond to upstream innervation and blood supply [11]. Mitochondrial dysfunction disrupts adipocyte metabolic flexibility, contributing to metabolic complications such as insulin resistance, obesity, and type 2 diabetes. Consequently, understanding the molecular mechanisms behind adipocyte mitochondrial dysfunction may offer potential strategies for preventing or delaying the onset of full-blown metabolic disorders through therapeutic intervention [12]. In this context, the research articles and reviews featured in the second edition of this Research Topic provide new insights into the pathogenesis, molecular pathways, and the beneficial effects of novel and safe treatments for metabolic diseases linked to adipose tissue dysfunction, as well as a deeper understanding of the molecular mechanisms involved in adipocyte mitochondrial dysfunction.

Among the cellular processes contributing to mitochondrial dysfunction, age-related adipose tissue senescence has emerged as a key factor in the development of comorbidities linked to metabolic disorders [13]. Cellular senescence alters various functional aspects of adipose tissue, leading to metabolic disturbances through impaired adipogenesis, increased inflammation, and abnormal adipocytokine production. These changes, in turn, trigger systemic inflammation and insulin resistance in metabolically active tissues, accelerating the decline of physiological functions [14]. Consequently, interventions that delay or prevent adipose tissue senescence could offer a new therapeutic approach to extending health and lifespan [15].

Additionally, in recent years, intermittent cold exposure (ICE) has gained attention as a method for improving mood, enhancing immune function, and reducing inflammation. ICE has also been proposed as an intervention to combat obesity and obesity-induced metabolic syndrome by activating BAT and altering WAT’s metabolic function, ultimately increasing energy expenditure. However, recent studies indicate that ICE does not consistently reduce body weight or fat mass, though it positively influences the metabolic consequences of obesity, such as improving glucose tolerance and insulin signaling. Furthermore, ICE consistently increases BAT activity and promotes the transition from WAT to beige adipose tissue. Interestingly, combining ICE with exercise does not appear to offer additional benefits, at least when exercising during ICE sessions [16].

Moreover, recent research has identified exposure to particulate matter, such as diesel exhaust particles (DEPs), as a factor contributing to metabolic disorders, including obesity, metabolic syndrome, and type 2 diabetes mellitus. A recent study by Warren and colleagues revealed that animals exposed to DEPs showed an increase in fat mass and a pronounced shift towards adipocyte hypertrophy. This study also found significantly higher expression of inflammatory markers in adipose tissue and substantial changes in adipose mitochondrial bioenergetics, including a two-fold increase in O_2_ consumption and, consequently, elevated reactive oxygen species generation [17].

Krupka et al. explored the effects of COVID-19 vaccination and infection on adipose tissue profiles. Their findings revealed that COVID-19 infection is linked to a reduction in average adipocyte size, with the impact being significantly more pronounced in subcutaneous adipose tissue compared to visceral adipose tissue, primarily due to immune system-related processes. Additionally, the infection led to a significant increase in the circulating levels of adiponectin, interleukin 6 (IL-6), and carbonic anhydrase 5A (CA5A), all of which are associated with obesity and glucose metabolism disorders. These results underscore the heightened vulnerability of individuals with obesity and type 2 diabetes to COVID-19, highlighting the need for targeted interventions to mitigate its effects in these people [18].

Among the external factors influencing adipose tissue, the purine nucleotide Inosine 5′-monophosphate (IMP), essential for organisms and known for its role in enabling animals to sense umami flavors, has shown a significant impact. Zhang et al. demonstrated that daily consumption of IMP led to hyperlipidemia and an increase in body fat percentage. This was associated with elevated expression of acetyl-CoA carboxylase 1 (ACC1) and phosphorylated acetyl-CoA carboxylase 2 (ACC2) in hepatocytes, as well as the promotion of adenosine 5′-monophosphate-activated protein kinase (AMPK) phosphorylation. ACC1 promoted the conversion of acetyl-CoA into triglycerides (TGs), which were then transported out of hepatocytes to prevent non-alcoholic fatty liver disease (NAFLD), leading to an acetyl-CoA deficiency in the liver. The increase in phosphorylated ACC2 facilitated the entry of fatty acids into mitochondria for conversion into acetyl-CoA via the fatty acid β-oxidation pathway, resulting in a fatty acid deficiency. Consequently, the liver increased its absorption of exogenous fatty acids, which were converted into TGs, contributing to lipohyperplasia and adipose metabolic dysfunctions [19].

Adipose tissue dysfunction can also contribute to obesity. Recently, advancements in genetic engineering have led to new precision medicine approaches targeting adipose tissue for anti-obesity therapy. These innovative strategies include gene therapy and cell-based treatments, such as adipose-derived mesenchymal stem cells (ADMSCs). Promising results from animal models and clinical trials with modified cells have shown their effectiveness in promoting weight loss, altering adipose tissue composition, and improving related comorbidities like diabetes and NAFLD. As a result, modified ADMSCs are emerging as a promising strategy for treating obesity and its associated conditions [20,21].

Previous studies have shown that individuals with obesity and related diseases tend to have lower concentrations of carotenoids, along with an increasing number of complications, suggesting that these molecules may offer protective effects against these conditions [22]. Supporting these findings, Queiroz and colleagues demonstrated that gelatin-based nanoparticles (EPG) loaded with a carotenoid-rich crude extract (CE), in Wistar rats with chronic systemic inflammation induced by a high glycemic load diet, led to reduced food intake and body weight, lower IL-6 and leptin levels, and an increase in multilocular adipocytes. These results suggest that EPG could serve as a nutraceutical for adjuvant therapy in treating inflammatory diseases associated with adipose tissue accumulation [23].

In conclusion, this Research Topic highlights that adipose organ senescence as well as the exposure to intermittent cold, diesel exhaust particles, COVID-19, and IMP are factors that can cause metabolic disorders and adipocyte mitochondrial dysfunction. Finally, the possibility to use new therapeutic options such as the EPG loaded with CE and the ADMSCs for treating obesity, and its comorbidities, was demonstrated. Overall, all of these fascinating results suggested that advances in understanding the molecular basis of obesity and its metabolic complications have given rise to new precision medicine approaches targeting adipose tissue as an anti-obesity therapy.
